# Using data envelopment analysis to measure the extent of technical efficiency of public health centres in Ghana

**DOI:** 10.1186/1472-698X-8-11

**Published:** 2008-11-20

**Authors:** James Akazili, Martin Adjuik, Caroline Jehu-Appiah, Eyob Zere

**Affiliations:** 1 Navrongo Health Research Centre, P.O. Box 114, Navrongo, Upper East Region, Ghana; 2 Policy, Planning, Monitoring and Evaluation division of Ghana Health Service, Accra, Ghana; 3 World Health Organization, P. O. Box 30390, Lilongwe, Malawi

## Abstract

**Background:**

Data Envelopment Analysis (DEA) has been used to analyze the efficiency of the health sector in the developed world for sometime now. However, in developing economies and particularly in Africa only a few studies have applied DEA in measuring the efficiency of their health care systems.

**Methods:**

This study uses the DEA method, to calculate the technical efficiency of 89 randomly sampled health centers in Ghana. The aim was to determine the degree of efficiency of health centers and recommend performance targets for the inefficient facilities.

**Results:**

The findings showed that 65% of health centers were technically inefficient and so were using resources that they did not actually need.

**Conclusion:**

The results broadly point to grave inefficiency in the health care delivery system of public health centers and that significant amounts of resources could be saved if measures were put in place to curb the waste.

## Background

A recent critical review of the Health Sector Reforms in Sub-Saharan Africa points to the fact that besides the issue of ever diminishing financial inflows to the health sector, poor quality of health care, mainly occasioned by a variety of inefficiencies at all levels of health care delivery is one of the most important concerns which has precipitated a number of reform initiatives and strategies in nearly all the developing countries [1]

There is also a growing concern among policy makers and planners that health services are not being delivered with utmost efficiency. In 2002, government in about 65% of the 46 countries in the WHO Africa Region spent less than US$ 10 per capita per year [2]. Evidence from the Africa Region indicates that the problem of scarcity of resources is also compounded with technical inefficiency that leads to wastage of the available meager resources [2]. In 2006, cognizant of the technical inefficiency plaguing the African health systems, Ministers of Health of the African Union Member States undertook to institutionalize efficiency monitoring within the national health information systems [3].

Coupled with this recognition, there is a realization among policy makers that increased funding alone will not and cannot solve the problem. From a strict sustainability perspective, it can be argued that most African countries are approaching or have already reached their upper limit in terms of increasing real financial resources allocated to the health sector. Given the escalating disease burden and the limited ability of governments, private and donor funds to meet this burden, the issue of health system sustainability has gained prominence in policy debates about finding a solution. These concerns are legitimate due to the magnitude of expenditure on health services, which account for as much as 5% of GDP and between 5% to 10% of government expenditures in developing countries, though this falls below the Abuja target of 15% of government expenditure allocated to the health sector [4,5]

Having provided rather generously for the creation and running of health centres, the Ghana government, international organizations and donors are beginning to question the performance of health centres [6]. Do health centres produce their outputs using the minimum amount of inputs feasible? Are there any inefficiencies related to the size of a health centre (too large or too small)? If all health centres operate efficiently, what are the possible efficiency savings? What are the lessons that can be drawn from the efficient health centre that are worth emulating by those that are inefficient so as to improve the efficiency of health centres and maximize efficiency savings? It is evident from these concerns that there is a knowledge gap as to the level of efficiency of health centres in the overall delivery of health services. Additionally the concern is also whether the volume or quality of services could be maintained by improving on the efficiency of health care delivery in health centres, in the face of current dwindling resources in developing countries.

To enhance the efficiency of health centers, planners need to develop methods to tackle the problems of accessibility, acceptability, intensity of use and compliance with medical instructions, quality of care, recurrent costs and community ownership [7]. To develop these methods, planners need prior knowledge of the efficiency levels in the health centers. Unfortunately there is limited literature on efficiency measures of health centers especially in developing countries and particularly in Africa and that must have informed the World Health Organization (WHO) Africa office to call for vigorous research on the efficiency of the health sector.

### Brief country profile

Ghana is located on West Africa's Gulf of Guinea only a few degrees north of the Equator. It lies between longitudes 3°15' W and 1°.12' E, and latitude 4°.44'and 11°.15' N. The country is bordered to the west by La Cote d'Ivoire, east by the Republic of Togo, Burkina Faso to the North and to the South by the Gulf of Guinea [8]

For administrative purposes, Ghana is divided into ten regions. The country is further divided into one hundred and thirty-eight administrative districts. Each of the district assemblies is headed by a nominated and approved District Chief Executive (DCE). The districts are further divided into sub-districts and units. The division of the country into regions, districts, sub-districts and units correlates with the health sector division in the provision of health services such that health centres are the highest health care facilities operating at the sub-district level.

Ghana's population was estimated to be about 21 million for 2007 with an annual growth rate of 1.3% [9]. The population density per square mile is estimated at 227. The Ghana Demographic and Health Surveys report that TFR declined from an average of 5.5 live children born per woman in 1993 to 4.2 children in 2003. In 2003, Infant and under-five mortality rates are estimated to have worsened to 64 and 111 deaths respectively per 1,000 live births compared to 57 deaths and 108 deaths in 1998 [9].

Ghana's economy is dominated by agriculture, which contributes over 42% of the Gross Domestic Product (GDP), followed by the service sector (38%). About 50 percent of the population relies on agriculture for their income. Ghana has a GDP per capita income of US$538, and is heavily aid-dependent and highly indebted to external creditors [10]. The country spends a total of US$252 million (4.2% of the GDP of US$ 6 billion) annually on health. About 53.5% of this expenditure is incurred by government and 46.5% by the household through out-pocket expenses. The total per capita expenditure on health at an average exchange rate is US$11 [11].

Health care delivery in Ghana is provided by both the public and private sectors, with the public sector organized according to national (2 teaching hospitals), regional (10 regional hospitals), district (281 district public and other hospitals), sub-district (622 public health centres) and community levels (1658 CHPS and maternity homes) [12], CHPS or Community-based Health Planning and Services is a programme for transforming clinic based primary health care to community-based health services. Out of the 281 district and other hospital, over 50% are private or mission hospitals. However these are heavily supported by government through staff salary and other facilities. They provide both outpatient and inpatient general services. At the sub-district level where health centres are the highest health facilities and first line of referral to the formal health from the community clinic and maternity homes, over 98% of them are public or belong to government. In other words, mission or private sector participation in the operation of health centres is very low. The Ministry of Health (MOH) is the central government agency responsible for oversight control by concentrating on sector-wide policy development, financing, regulation, monitoring and evaluation using its agencies including Ghana Health Service (GHS) which is an executing agency responsible for health service delivery. However despite the strategically dispersed location of health centres in the country, the teaching, regional and district hospitals still have to contend with high outpatient and other primary health related cases which could be managed at that level. This phenomenon raises doubts on the efficiency of health centres.

### Measurement of efficiency in Africa

Data Envelopment Analysis (DEA) has been extensively used in Asia [13], the Americas [14,15] and Western Europe [16-19] to shed light on the efficiency of various aspects of national health systems. In Africa, the application of DEA in the health sector has been quite limited. So far, the approach has been applied to health facilities in only few countries, i.e. a study of 155 primary health care clinics in Kwazulu-Natal province in South Africa found 70% of them to be technically inefficient [20]. A similar study of 32 public health centres in Kenya revealed that 56% of them were technically inefficient [21]. Kirigia (2002) also assessed the technical efficiency of 54 public hospitals (which are higher level of health care) using the DEA application in Kenya and found that 26% (14) of the hospitals were technically inefficient [22]. The study singled out the inefficient hospitals and provided the magnitudes of specific input reductions or output needed to attain technical efficiency. An assessment of technical efficiency of 30 district hospitals in Namibia was carried out in 2006 using DEA and the findings were similar to that of public hospitals in Kenya [22]. The average technical efficiency was less than 75% [23]. Another study in Sierra Leone revealed that 59% of the 37 peripheral health units in Pujehun district were technically inefficient [24]. A recent technical efficiency study using DEA in Zambia of 20 hospitals revealed average efficiency of 64% implying that the 17 inefficient hospitals could lower their cost by 36% and still achieve their current levels of output [25]. A pilot study of 21 public health centres and 21 hospitals was carried out five years ago in Ghana [26] and the results shows that 18% of the health centres were technically inefficient, According to the paper, the sample of the health centres was too small (3.7%) that the results could not be generalised for the whole country and so suggested further studies on the technical and allocative efficiency of health centres. It is important to note that the assessment of the efficiency ought to be more prevalent in low-income countries like Ghana in order to optimise health benefits from the available meagre health sector resources.

## Methods

### Data

The total number of public health centres in Ghana was 622 in 2004 [12] and using an expected inefficiency rate of the health centres to be 20% (based on the previous pilot study by Osei et al (2005) in Ghana), a precision of 8% (based upon a power calculation) at the 95% confidence level we calculated the required sample size as 84. Since this was a nation-wide survey, we expected about 10% missing/non-response in the collection of the data and the expected sample size came to 92 health centres. After cleaning and eliminating health centres with missing data, the sample size came to 89. The two researchers who were trained on how to collect the data visited each of the health centres in the sample in 2005 and reviewed their 2004 inputs and outputs records and a structured form was used to collect the inputs and output data. Inputs in the health centre production are classified as human resources (clinical and non-clinical staff), expenditure on drugs and other consumables and number of beds and cots. Outputs were categorized into outpatient visits, number of antenatal care visits, number of deliveries, number of children immunized, number of family planning visits. These inputs and outputs were use to estimate the technical efficiencies of the health centres. The instruments were pre-tested for consistency and accuracy before actual data collection. Data collection was preceded by a certification from the Ethical Review Committee of the Ghana Health Service. Consent was sought at each health facility before data collection. Supervision was conducted by the Principal Investigator to ensure that data were properly or scientifically collected. Data collected were entered using Epi Info™ 3.3, and the technical efficiency scores were computed using Data Envelopment Analysis programme, version 2.1 (DEAP 2.1).

### Selection of inputs and outputs data

The selection of inputs and outputs for a DEA study needs careful attention as it may affect the distribution of technical efficiency. Improved health status is the ultimate output of a health system. However, improved health status is influenced by a host of factors some of which are outside of the domain of the health sector. Furthermore, measuring improvements in health status accurately is fraught with difficulties. Health centres and other health care organizations rarely collect information on health outcomes routinely. Therefore output is measured by intermediate health services that ostensibly improve health status [11]. Health centres in Ghana deliver outpatient curative and preventive care. They have a strong bias towards health promotion and disease prevention [26]. The issue of case mix and variation in the quality of care is not expected to be a problem, as health centres are standardized in terms of their staffing and other resources and the types of curative and preventive programmes that they run. Inputs in health centre production can be classified as labour (clinical and non-clinical), capital (proxied by the number of beds used for emergency cases and child deliveries) and supplies including pharmaceuticals. The choice of inputs and outputs for the DEA analysis was guided in part by the previous DEA health care studies in the African Region and availability of data [23,25,26]. The inputs and output selected include the following.

#### Inputs

Input 1: Number of non clinical staff including labourers

Input 2: Number of clinical staff

Input 3: Number of beds and cots

Input 4: Expenditure (in local currency call cedi) on drugs and supplies. The inter-bank exchange rate of the cedi to the dollar was ¢8,500 to 1 US$ at the time of the study.

#### Output

Output 1: General outpatient visits

Output 2: Number of antenatal care visits

Output 3: Number of deliveries

Output 4: Number of children immunised

Output 5: Number of family planning visits

### Efficiency and DEA Analytical framework

The basic premise underlying the concept of efficiency is that no output can be produced without resources (inputs) and that these resources are limited in supply. From this, it also follows that there is a limit to the volume of output (commodities) that can be produced.

There are two basic measures of efficiency: allocative and technical efficiency. Allocative efficiency refers to how different resource inputs are combined to produce a mix of different outputs [27]. Technical efficiency on the other hand is concerned with achieving maximum outputs with the least cost. Overall efficiency measures the combined effect of allocative and technical efficiency [27].

In order to measure efficiency a norm must be specified. The norm set for measuring *technical efficiency *is that the minimum amount of resources should be used for a given level of output or, alternatively, the maximum amount of output that should be produced for a given level of resource use. If more resources than necessary are used to produce a given amount of output, this implies a waste of resources and therefore inefficiency. Equally, the difference in the amount of output that could have been produced from a given amount of resources and the amount of output that was actually produced can be used as a measure of technical inefficiency [28]. Technical inefficiency is thus a matter of degree depending upon how much unnecessary resources have been used. The size of a health centre may sometimes be a cause for inefficiency. A health centre may be too large for the volume of activities that it is conducting; and therefore may experience *inefficiencies of scale*. In the presence of inefficiencies of scale, a health centre is inefficiently large, unit costs increase as the scale of production increases. On the other hand, a health centre may be too small for its level of operation, and thus experience efficiencies of scale.

Until recently, the traditional methodology for measuring efficiency in economics (including health economics) has been the production frontier approach based on the principles of statistics and econometrics [28]. These functions, which are estimated to determine efficiency, are also known as stochastic frontier models (SFM). During the recent few decades, however, an alternative methodology to the stochastic frontier approach (SFA) has been developed and its application has grown rapidly over the years. This methodology has come to be known as the Data Envelopment Analysis (DEA) [28]. It has been found that there are several compelling methodological and practical advantages for using DEA over the stochastic frontier models. DEA accommodates multiple inputs and multiple outputs in a single measure of efficiency than the SFA and has become the dominant approach to efficiency measurement in health care and in many other sectors of the economy [16]. DEA does not impose a specified functional form to model and calculate the efficiency of a decision making unit (DMU). Unlike the parametric frontier models therefore, DEA does not suffer from the problem of model mis-specification, which has the potential of providing misleading results [28]. In addition, Unlike SFA, DEA does not suffer from the problems of multicollinearity and heteroscedasticity. DEA gives a measure of efficiency that is empirically obtainable in a given scenario (given available resources, institutional set-up, etc). Hence we can compare the efficiency of individual health centres realistic benchmarks.

On the other hand, DEA estimation can only tell how well a DMU or health centre (in our case) is doing compared to its peers but not compared to a "theoretical maximum". in other words since DEA gives a relative measure of efficiency it has the potential of justifying inefficiency i.e. even those that appear to be efficient in the sample might actually be inefficient in absolute terms. This problem can, however, be minimized by using a large sample data set. Another limitation or disadvantage is that since DEA is a non parametric technique, statistical hypothesis testing is difficult to do. Also since DEA is an extreme point technique, noise such as measurement errors can cause significant problem. Further overview of the DEA model is presented below

For assessing differences in the productive efficiency of health centres, we use DEA, a mathematical programming based method that converts multiple input and output measures into a single summary measure of productive efficiency. DEA is based on relative efficiency concepts proposed by Farrell but Charnes et al (1994) extended and developed Farrell's approach. DEA can be said to utilize an extended concept of Pareto efficiency [28].

Following Charnes et al (1978) the technical efficiency of health centres as the maximum of a ratio of weighted outputs to weighted inputs subject to the condition that the similar ratios for every health centre be less than or equal to unity. This is done by solving the following fractional programming problem

(1) $$\begin{array}{l} Max\;h_{0}=\frac{\sum_{r=1}^{s}u_{r}y_{rjo}}{\sum_{i=1}^{m}v_{i}y_{ijo}}\\ \begin{array}{cc} \text{Subject to} & \\ & \begin{array}{cc} \frac{\sum_{r=1}^{s}u_{r}y_{rj}}{\sum_{i=1}^{m}v_{i}x_{ij}}\leq 1, & j=1,...j_{0},...n \end{array}\\ & \begin{array}{ccc} u_{r}\geq 0,r=1,...,s & \text{and} & v_{i}\geq 0,i=1,...,m \end{array} \end{array} \end{array}$$

The terms *y*_*rjo *_and *x*_*rjo *_represent the amount of output *r *and the amount of input *i *for the unit *j*_0_. Optimization is performed separately for each unit to compute an optimal set of weights (*u*_*r*_, *v*_*i*_) and efficiency measure *h*_0_. The method chooses values of *u*_*r *_and *v*_*r *_which are most favorable to the unit that is being studied. As a consequence, a unit that is superior to all others on any single output-input ratio will be rated efficient.

The standard DEA model, the relative efficiency of production unit is defined as the ratio of the sum of its weighted outputs to the sum of its weighted inputs. The weights have been determined so as to show the production unit at the maximum relative efficiency.

In the study we will adopt the input oriented-based approach because decision making units (Health centres) have better control over inputs than outputs hence our interest in the input based approach. This approach is also more popular in terms of usage than the output oriented approach [29,30,20,21,23,25]. The model in (1) is a fractional programming model, which can be converted into the following linear forms (models 2 and 3) so that the methods of linear programming can be applied.

### Constant Returns to Scale (CRS) model

The constant returns to scale model assumes a production process in which the optimal mix of inputs and outputs is independent of the scale of operation. The following CRS model measures overall technical efficiency for each of the sample health centre. The objective function is to maximize the efficiency score *h*_0 _for health centre *j*_0_, subject to the constraints that no health centre will be more than 100% efficient and the coefficient values are positive and non-zero, when the same set of *u *and *v *coefficients (weights) are applied to all other health centres being compared.

(2) $$\begin{array}{l} Max\;h_{0}=\sum_{r=1}^{s}u_{r}y_{rj_{0}}\\ \begin{array}{ll} \text{Subject to} & \sum_{i=1}^{m}v_{i}x_{ij_{0}}=1\\ & \begin{array}{cc} \sum_{r=1}^{s}u_{r}y_{rj}-\sum_{i=1}^{m}v_{i}x_{ij}\leq 0 & j=1,...,n \end{array}\\ & \;u_{r},v_{i}\geq 0 \end{array} \end{array}$$

### Variable Returns to Scale (VRS) model

The VRS model, though similar to the CRS model, measures pure technical efficiency and returns to scale for each of the sample health centres. Scale efficiency can be measured by dividing the CRS efficiency score by the VRS efficiency score. From the VRS model, it is possible to analyze whether a health centre's production indicates increasing return to scale, constant return to scale, or decreasing return to scale by the sign of the variable *z*_*jo*_. Increasing returns to scale exists if the value of *z*_*jo *_is greater than zero (*z*_*jo *_> 0), constant returns to scale if the value of *z*_*jo *_is equal to zero (*z*_*jo *_= 0), and decreasing returns to scale if the value of *z*_*jo *_is less than zero (*z*_*jo *_< 0). Thus, we can analogize the existence of efficiencies of scale similar, confirm the most productive scale size (minimum efficient scale) of a health centre and estimate the number of health centres operating at the efficient scale.

(3) $$\begin{array}{l} Max\;h_{0}=\sum_{r=1}^{s}u_{r}y_{rj_{0}}+z_{j0}\\ \begin{array}{lll} \text{Subject to} & \sum_{i=1}^{m}v_{i}x_{ij_{0}}+z_{j0}=1 & \\ & \sum_{r=1}^{s}u_{r}y_{rj}-\sum_{i=1}^{m}v_{i}x_{ij}+z_{j0}\leq 0 & j=1,...,n\\ & & u_{r},v_{i}\geq 0 \end{array} \end{array}$$

The paper concentrated on the VRS model. This is so because the VRS model isolates the pure technical efficiency component and scale efficiency which related to the size or structure of the decision making unit (DMU). Health centres that are overall efficient exhibit constant returns to scale. The size of a Health centre may sometimes be a cause for inefficiency. A health centre may be too large for the volume of activities that it is conducting; and therefore may experience *inefficiencies of scale*. On the other hand, a health centre may be too small for its level of operation, and thus experience efficiencies of scale. Inefficiency due to congestion refers to too many inputs (staff, funds, drugs, etc) leading to decreased output or what is commonly known as inefficiencies of scale which to some extend are realistic assumption for a developing country like Ghana where political and other irrational reasons affect the establishment of facilities such health centres, schools etc. It is important to point out that this study does not attempt to address allocative efficiency in the paper, as it was difficult to get accurate input prices. The study also does not address issues of productivity, due to lack of appropriate panel data.

## Results

**Figure 1:**
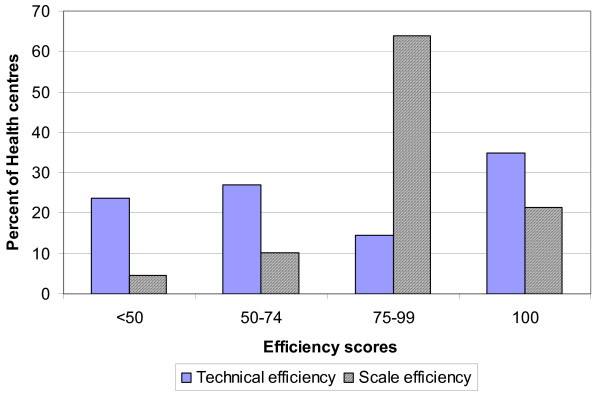
Distribution of technical and scale efficiency scores.

Table 1 presents the mean and standard deviations of the inputs and output variables of the 89 public health centres. Table 2 also presents the technical efficiency scores and the scale efficiency levels of the 89 health centres (HC). It is important to note that efficiency scores range from 0 (totally inefficient) to 100% (efficient). Out of the 89 health centres in the analysis 31 (35%) were technically efficient whereas the remaining 58 (65%) where technically inefficient. Among the inefficient health centres 21 (24%) had a technical efficiency score of less than 50%, 24 health centres (27%) between 50 and 74% (see Figure 1). The inefficient health centres had an average TE score of 57% and a standard deviation of 19%. This implies that on average they could reduce their utilization of all inputs by about 43% without reducing output.

**Table 1:** Means (M) and standard deviations (SD) of efficient and inefficient health centres

	**Efficient health centres**		**Inefficient health centres**	
**Input**	**M**	**SD**	**M**	**SD**
Input 1: Number of non clinical staff	3.5	2.6	4.2	2.3
Input 2: Number of clinical staff	5.3	4.1	5.2	2.6
Input 3: Number of beds and cots	5.6	5.5	7.3	4.9
Input 4: Expenditure on drugs and supplies	33,290,526	33,480,387	39,369,572	41,513,906
				
**Output**				
Output 1: General outpatient visits	5,183	5,123	3,783	3,239
Output 2: Number of antenatal care visits	632	907	424	378
Output 3: Number of deliveries	165	191	110	108
Output 4: Number of children immunised	2,250	2,907	1,307	1,856
Output 5: Number of family planning visits	1,122	1,145	631	455

**Table 2:** Technical and scale efficiency scores for health centres

**Health centre**	**Technical Efficiency score (%)**	**Scale efficiency (%)**	**Health centre**	**Technical Efficiency score (%)**	**Scale efficiency (%)**
Abofour	76.1	88.1	Kojokper	100.0	100.0
Abore	33.4	98.9	Kona	48.5	100.0
Abutia	100.0	77.0	Kpedze	76.2	96.4
Adahlu	49.7	93.0	Kpetoe	100.0	81.7
Aagorve	38.6	98.8	Kumawu	78.3	84.0
Ahenkro	94.5	95.2	Kunchogu	100.0	57.9
Akomadan	88.0	68.6	Kundungu	45.4	94.2
Antoakro	67.5	92.8	Kwanuoma	79.3	70.3
Anyinasu	100.0	47.0	Kyekyew	28.6	88.7
Azolokpu	35.9	83.0	Loggu	71.6	98.7
Banka	35.3	90.3	Mamfo	63.6	90.0
Betiako	65.0	91.2	Matse	47.3	48.4
Binduri	100.0	100.0	Mpasaso	67.9	97.9
Boamang	50.4	89.0	Nabugube	100.0	88.1
Boanim	64.4	97.7	Nabulo	31.2	89.8
Bolgacen	100.0	100.0	Namoo	85.6	88.5
Bompata	58.2	99.7	Nangodi	100.0	100.0
Bomso	100.0	100.0	Nanvilli	100.0	100.0
Bongosoe	100.0	100.0	Nnadieso	80.0	87.8
Bugri	54.9	98.6	Nyive	100.0	91.1
Busa	100.0	100.0	Ofoase	35.6	97.9
Bussie	100.0	77.8	Paga	95.8	67.3
Charia	31.7	98.0	Pokukrom	100.0	100.0
Charikpo	100.0	100.0	Pusiga	100.0	100.0
Chiana	100.0	85.9	Pwalugu	100.0	100.0
Chuchuliga	63.2	99.4	Semum	52.8	90.0
Dapuori	52.6	67.1	Shama	52.9	99.3
Dodome	71.1	45.6	Shia	39.8	98.8
Dorimon	100.0	100.0	Subriso	57.9	83.9
Dwendwenas	30.9	75.7	Suromu	38.5	97.6
Edubia	56.0	69.3	Tetrefu	100.0	72.5
Fasin	100.0	100.0	Tetrem	20.8	99.7
Fian	28.8	95.1	Trabuom	100.0	100.0
Foase	45.0	97.5	Trede	100.0	41.5
Fumbisi	88.1	96.7	Tsito	100.0	100.0
Gwollu	100.0	89.1	Vea	100.0	100.0
Helfi	36.9	95.6	Walembel	100.0	100.0
Issa	40.3	99.8	Wechiau	60.1	99.0
Jachie	62.3	87.5	Wiaga	100.0	90.3
Jamasi	64.7	73.6	Workambo	76.8	89.4
Jang	57.1	99.7	Yaala	51.0	88.7
Jeffisi	100.0	59.0	Zongoire	89.7	88.5
Kaleo	56.2	98.6	Zorko	68.0	92.9
Kanjarga	47.0	92.9	Zuarungu	69.4	77.1
Kneast	76.6	81.4			

On the other hand out of the 89 health centres analysed 19 (21%) were scale efficient whereas the remaining 70 (79%) where scale inefficient. Among the inefficient health centres 4 (4.5%) had a scale efficiency score of less than 50%, 9 health centres (10%) between 50 and 74%, 57 (64%) between 75 and 99 (see Figure 1). The inefficient health centres had an average scale score of 86% (with a standard deviation of 14%); implying there is potential for increasing total outputs by about 14% using the existing capacity/size.

Table 3 also displays the inputs reductions and/or output increases needed to make individual inefficient health centres efficient. Table 4 also provides a summary of total input savings that would have resulted if the inefficient health centres were operating efficiently.

**Table 3:** Inputs reductions and/or output increases needed to make individual inefficient health centres efficient

**Inefficient health centres**	**Non-clinical staff**	**Clinical staff**	**Beds and cots**	**Recurrent expenditure**	**Out-patients**	**Immunis-ation**	**Family planning**	**ANC**	**Delivery**
Paga	4	9	6	51,108,079	15,990	8,426	1,685	1,423	303
Ahenkro	4	3	4	42,543,020	7,417	1,568	746	584	240
Zongoire	2	3	2	13,307,795	3,621	3,020	1,037	245	71
Fumbisi	3	6	4	38,571,363	7,272	5,261	990	1,236	472
Akomadan	7	4	7	92,717,275	7,895	1,471	651	1,677	1,006
Namoo	3	5	3	37,013,661	10,807	2,738	1,198	774	153
Nnadieso	3	2	3	16,600,000	3,209	981	485	509	132
Kwanuoma	2	2	3	12,466,000	1,479	559	297	230	126
Kumawu	6	7	6	73,724,832	17,382	4,187	1,306	1,411	599
Workambo	4	6	4	39,998,716	11,867	9,246	972	696	334
Kneast	4	10	4	29,855,715	8,789	10,290	2,350	1,020	202
Kpedze	3	4	5	7,520,000	2,522	542	1,565	371	55
Abofour	7	7	5	68,759,850	7,230	2,564	1,544	1,423	810
Loggu	3	4	5	12,118,005	3,396	1,039	1,251	779	66
Dodome	1	2	1	6,970,000	1,214	156	335	162	41
Zuarungu	4	10	4	33,105,590	9,496	8,505	2,652	874	230
Zorko	3	7	3	23,377,608	6,207	4,942	1,335	998	318
Mpasaso	2	5	4	23,114,164	4,157	2,167	3,136	674	70
Antoakro	2	3	4	20,949,800	3,413	1,078	1,140	588	209
Betiako	5	3	2	21,959,614	5,094	1,226	901	571	140
Jamasi	6	9	5	60,174,217	23,829	6,065	1,775	1,224	317
Boanim	3	2	7	33,591,124	4,824	330	530	941	283
Mamfo	3	3	2	14,311,650	2,144	1,439	2,012	310	27
Chuchuliga	2	6	7	24,090,119	7,917	5,310	1,229	794	126
Jachie	4	6	8	62,867,862	10,236	2,162	1,492	3,205	342
Wechiau	3	3	4	16,560,350	5,524	2,645	720	830	95
Bompata	3	5	6	33,714,200	12,797	2,613	1,083	1,067	206
Subriso	2	3	2	11,294,000	1,846	586	1,637	244	42
Jang	2	4	3	16,845,992	3,598	555	1,978	662	151
Kaleo	4	6	4	21,124,022	9,004	4,205	1,510	499	151
Edubia	5	7	7	57,112,489	15,602	3,649	2,154	2,066	233
Bugri	3	6	6	49,732,000	7,641	9,867	1,018	1,405	482
Shama	2	5	5	29,226,500	5,837	4,359	838	907	378
Semum	2	4	3	11,985,850	2,948	996	542	510	225
Dapuori	1	2	4	11,370,600	3,017	195	521	544	52
Yaala	2	3	3	9,200,828	2,924	1,058	906	326	71
Boamang	4	7	5	49,903,750	13,700	7,261	1,311	1,024	466
Adahlu	3	3	2	10,545,264	2,578	531	1,046	414	117
Kona	4	4	7	53,357,727	10,072	1,848	835	1,285	482
Matse	2	2	2	5,518,858	1,388	406	264	144	45
Kanjarga	2	4	5	20,544,530	7,845	4,002	601	996	130
Kundungu	2	3	4	14,366,000	3,630	371	963	859	53
Foase	4	7	6	60,180,267	14,140	7,961	1,249	1,699	478
Issa	3	4	5	32,297,027	7,170	1,392	2,363	953	144
Shia	3	4	6	16,514,048	4,940	834	1,893	629	85
Agorve	2	5	3	22,675,250	4,878	954	2,605	748	176
Suromu	4	7	4	44,630,570	14,899	3,679	1,294	1,461	193
Helfi	3	4	3	12,298,900	2,345	528	2,094	376	84
Azolokpu	2	2	5	9,400,000	3,246	359	435	521	61
Ofoase	3	3	6	34,056,026	7,361	1,748	660	1,036	222
Banka	2	4	3	26,294,600	3,893	3,830	605	547	342
Abore	3	6	3	35,243,950	7,992	2,289	1,025	1,031	458
Charia	2	4	5	20,330,624	6,935	2,559	751	800	202
Nabulo	2	3	3	10,068,320	2,759	942	997	417	77
Dwendwenas	2	2	3	13,516,870	3,068	897	524	463	62
Fian	3	3	5	8,038,815	3,282	282	667	462	66
Kyekyew	4	7	4	41,176,000	7,986	3,856	1,397	1,297	493
Tetrem	3	4	4	35,350,000	5,682	1,202	1,726	851	317

**Total**	**180**	**266**	**248**	**1,705,290,285**	**397,936**	**163,734**	**70,827**	**49,793**	**13,513**

*Inter-bank exchange rate of the cedi (local currency) to the US dollar in 2004 was ¢8,500 to US$1

**Table 4:** Total input savings from inefficient health centres

**Type of input**	**Actual inputs use**	**Inputs that owe to be used**	**Input savings**
Non clinical staff	246	180	66
Clinical staff	299	266	33
Beds and cots	427	248	179
Recurrent expenditure	2,328,829,264	1,705,290,285	623,538,979 (US$73,357,53)

The average technical efficiency scores levels were also calculated according to the three broad division of the country, the northern, middle and coastal belts. In the sample the northern belt was calculated based on sample health centres from Upper East and Upper West regions; the middle composed of Ashanti and Volta regions and the coastal is made up of Greater Accra and Western regions. The estimates showed that average technical efficiency scores were highest amongst health centres in the coastal belt, followed by the northern belt. Health centres in the middle belt recorded the lowest average efficiency scores.

## Discussion

Public health centres support the Community-based Health Planning and Services (CHPS) and provide preventive, affordable, promotive, and basic curative care in localities inhabited mainly by the poor. Their location makes them critically important in the ongoing efforts to scale up pro-poor cost-effective public health interventions geared at achieving the health related Millennium Development Goals (MDGs) [11] and New Partnership for Africa's Development (NEPAD) health targets [31]. Thus, the importance of these close-to-client health facilities in all efforts to reduce the burden of disease and improve health conditions, especially in rural areas, cannot be overemphasized.

The results point to grave technical inefficiency in the Ghana health system especially at the lower level of care. The results of the 89 public health centres sampled shows that 65% of them are technically inefficient. The findings of this study are in line with other studies in sub-Saharan Africa, which indicate the wide prevalence of technical inefficiency [20,21,24,25]. According to Osei et al (2005) study in Ghana a sample of the health centres was too small (3.7%) that the results could not be generalised for the whole country and so suggested an expanded study in this direction. The current study samples over 14% of the public health centres in Ghana.

On average, health centres are using more inputs than they need to produce what they are currently producing. Put differently, health centres could increase on the number of outpatients, ANC registrants, deliveries and family planning services with the resources they have currently. However, since we do not expect health centres to go out and look for more patients or clients, in the name of increasing output, cost minimisation might be the noble objective to aspire to. In essence the operations and performance of health centres could be strengthened if resources are better utilised. The study clearly reveals a substantial amount of input savings, which could go a long way in injecting additional resources to the health system to address the backlog of inequities and/or further improve the quality of the available health care. For example, the efficiency saving (see Table 4) that could have been realized is US$73, 357, 53.

Table 3 provides the magnitudes by which specific inputs per inefficient health centre ought to be reduce. Equipped with this information, policy makers and health centres managers could proactively improve the efficiency of primary delivery by transferring clinic staff to more efficient health centres that will enhance the capacity of primary health sector to response to the needs of the people. They could also send non clinical staff to early retirement and the savings used to improve on the quality of the facilities. With regards to the beds and cots, transfer them to more efficient facilities; they can also sell them or enter into partnership with private providers to use them at the price which should not be less than the marginal cost

The study further reveals that the prevalent scale inefficiency is increasing returns to scale. In the presence of increasing returns to scale, expansion of outputs reduces unit costs. Because increasing the level of outputs requires an increase in the demand for health care which is beyond the control of the health centre's management, a merger of two centres in close geographic proximity is an option worth considering. However, this option may potentially pose some problems given the low density of population in some of the areas. Residents may even incur additional costs in travel expenditure and in delayed treatment of emergency cases. These potential problems may to some extent be minimized by pursuing vigorously the government current policy of Community-base Health Planning and Service (CHPS). In all these the equity implications must be taken in consideration

**Figure 2:**
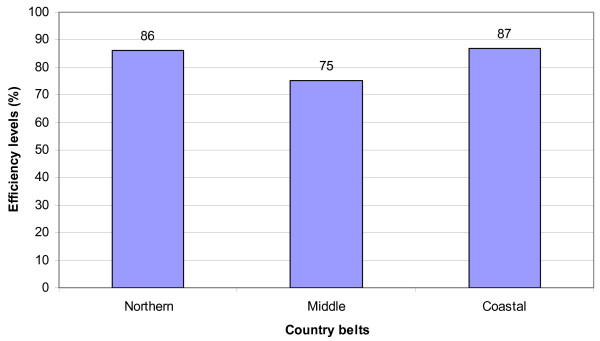
Distribution of efficiency scores by location (belts).

The current under five and maternal rates in Ghana is quite high given the targets of the MDGs by 2015. Currently Ghana and many African countries are strengthening and promoting CHPS to help improve their health indicators (especially on child and maternal mortality). Health centres which are referral points to community based health care efforts (community clinics and maternity homes) could help play a significant role in the desire to improve on health indicators towards meeting the MDG targets. However, health centres can play this role well if their efficiency levels are known and dealt with. The findings provide the bases upon which Government, policy makers and all other relevant stakeholders will target efforts to reduce the identified inefficiency of the health centres (see Table 3). In the case of Ghana and in this study, efforts will need to be directed to reducing inefficiency of health centres especially those at the middle belt of the country as indicated in figure 2.

The DEA analysis of the health centres could be factored into the monitoring of the health system in which case, it could be assessed every year. During the data collection at the health centres we also identified the problem of information capturing, storage and management and so it will be important that training in management information systems for all health centres to enable them produce timely and reliable information be instituted.

## Conclusion

The study has shown that only 35% of the health centres in Ghana are efficient and even though this findings is perfectly inline with other findings from other developing countries particularly from Africa [20-25], its implications with regards to health care provision (given the limited resources in the health sector) is of serious concern. Given that primary health care is an important driver in the health care system of most developing countries including Ghana, efforts are needed to making the health centres that are not operating on the frontier efficient.

The study has demonstrated that DEA is an essential tool for identifying the most and least efficient health centres, and strategies for saving resources/inputs and/or increasing output. We concur with Kirigia et al [22] and Boussifiane et al [32] that DEA can be used in identifying efficient operating practices and efficient strategies, setting targets/bench marks for relatively inefficient health centres, monitoring effects of health sector reforms on efficient over time, and resource allocation

It would have been interesting and relevant to unearth the causes of inefficient in the health centres unfortunately it was not possible to get complete and reliable data that could be used to unpack the causes of technical inefficiency using a second stage Tobit regression analysis. For further studies in this area we recommend an examination of the causes of inefficiencies in health centres. It will also be interesting to look at allocative efficiency which is closely related to technical efficiency and which warrants the collection of price data in addition. With good panel data for a sufficiently longer period of time it will be important and interesting to also do further research to estimate DEA-based Malmquist productivity index (MPI) to observe the changes in efficiency and those changes in productivity that are accounted for by technological change. This study is on public health centres, it will important to examine technical and allocative efficiency by ownership of higher level facilities in Ghana like the district and regional hospitals.

## Competing interests

The authors declare they have no competing interests. The study was supported with grants from the Ghanaian-Dutch for research and development and the sponsors had no involvement in the study design; in the collation, analysis and interpretation of the data; in the writing of the paper, and in the decision to submit the paper for publication.

## Authors' contributions

JA, MA and CJA contributed to the study design, analyzed the data and wrote the manuscript. ZE contributed to the study design and implementation and also critically read through the manuscript and restructured the paper. All authors read through and agreed for the paper to be submitted for publication.

## Pre-publication history

The pre-publication history for this paper can be accessed here:



## References

[B1] Leighton C, Makinen M (1999). Health sector reforms in Sub- Saharan Africa. Paper presented in a Workshop.

[B2] Kirigia JM, Preker A, Carrin G, Mwikisa C, Diarra-Nama AJ (2006). An overview of health financing patterns and the way forward in the WHO Africa Region. East Afr Med J.

[B3] Kirigia JM, Asbu EZ, Greene W, Emrouznejad A (2007). Technical efficiency, efficiency change, technical progress and productivity growth in the national health systems of continental African Countries. Eastern Africa Social Science Research Review.

[B4] World Bank (2004). The millennium development goals for health: rising to the Challenges.

[B5] Ghana Macroeconomics and Health (2005). Scaling-Up Health Investments for Better Health, Economic Growth and Accelerated Poverty Reduction.

[B6] World Health Organisation (1992). The role of health centres in the development of urban health systems.

[B7] World Health Organization (2000). The World Health Report. Improving performance of health systems Geneva.

[B8] Government of Ghana (2002). Ghana Poverty Reduction Strategy. An agenda for growth and prosperity Accra.

[B9] Ghana Statistical Service (2003). Ghana Demographic and Health Survey. Ghana Statistical Service and Macro International Carlverton.

[B10] World Health Organization (2002). The World Health Report. Geneva.

[B11] United Nations Development Programme (2003). Human Development Report. Millennium Development Goals: A compact among nations to end human poverty.

[B12] Ghana Health Services (2005). Facts and Figures. Policy, Planning, Monitoring and Evaluation.

[B13] Chang H (1998). Determinants of hospital efficiency: the case of central government- owned hospitals in Taiwan. Omega International Journal of Management Science.

[B14] Chattopadhy S, Ray CS (1996). Technical, scale, and size efficiency in nursing home care: a nonparametric analysis of Connecticut homes. Health Economics.

[B15] Shroff HFE, Gulledge TR, Haynes KE, O'neill MK (1998). Efficiency of long-term health care facilities. Socio-Economic Planning Science.

[B16] Giuffrida A, Gravelle H (2001). Measuring performance in primary care: econometric analysis and DEA. Applied Economics.

[B17] Ersoy K, Kavuncubasi S, Ozcan YA, Harris JM (1997). Technical efficiencies of Urkish hospitals: DEA approach. Journal of Medical Systems.

[B18] Jacobs R (2001). Alternative methods to examine hospital efficiency: data Envelopment analysis and stochastic frontier analysis. Health Care Management Science.

[B19] Linna M, Nordblad A, Koivu M (2003). Technical and cost-efficiency of oral health care provision in Finnish health centres. Soc Sci Med.

[B20] Kirigia JM, Sambo LG, Scheel H (2001). Technical efficiency of public clinics in Kwazulu-Natal province of South Africa. East Afr Med J.

[B21] Kirigia JM, Emrouznejad A, Sambo LG, Munguti N, Liambila W (2004). Using Data Envelopment Analysis to measure the technical efficiency of public health centres in Kenya. Journal of Medical Systems.

[B22] Kirigia JM, Emrouznejad A, Sambo LG (2002). Measurement of technical efficiency of public hospitals in Kenya: using data envelopment analysis. Journal of Medical Systems.

[B23] Zere E, Mbeeli T, Shangula K, Mandlhate C, Mutirua K, Tjivambi B, Kapenambili W (2006). Technical efficiency of district hospitals: Evidence from Namibia using Data Envelopment Analysis. Cost Effectiveness and Resource Allocation.

[B24] Renner A, Kirigia J (2005). Technical efficiency of health centers in Sierra Leone. African Health Economics Monitor.

[B25] Masiye F (2007). Investigating health system performance: an application of data envelopment analysis to Zambia hospitals. BMC Health services research.

[B26] Osei D, D'Almeida S, Melvill OG, Kirigia JM, Ayayi OM, Kainyu LH (2005). Technical efficiency of public district hospitals and health centres in Ghana: a pilot study. Cost Effectiveness and Resource Allocation.

[B27] Coelli TJ (1996). A guide to DEAP version 2.1: A Data Envelopment Analysis Programme. CEPA working paper 96/8, department of econometrics.

[B28] Charnes A, Cooper W, Lewin AY, Seiford LW (1994). Data Envelopment Analysis: Theory.

[B29] Zere EA, Addison T, McIntyre D (2000). Hospital efficiency in sub-Saharan Africa: evidence from South Africa. South African Journal of Economics.

[B30] Kirigia JM, Lambo E, Sambo LG (2000). Are public hospitals in Kwazulu-Natal Province of South Africa technically efficient. African Journal of Health Sciences.

[B31] New Partnership for Africa's Development (NEPAD) (2001). Human Development Programme. NEPAD Health Strategy Pretoria.

[B32] Boussofiane A, Dyson RG, Thanassoulis E (1991). Applied data envelopment analysis. Eur J Oper Res.

